# Antibiotic resistance in *Aeromonas hydrophila* associated with exposure to subtherapeutic levels of oxytetracycline

**DOI:** 10.3389/fmicb.2026.1794341

**Published:** 2026-05-08

**Authors:** Ju Zhang, Liqing Huang, Liang Zhong, Zhaoyang Wang, Celia Schunter, Sophie St-Hilaire

**Affiliations:** 1 Department of Public Health and Infectious Diseases, Jockey Club College of Veterinary Medicine and Life Sciences, City University of Hong Kong, Kowloon Tong, Hong Kong SAR, China; 2 College of Animal Sciences and Technology/College of Veterinary Medicine, Huazhong Agricultural University, Wuhan, Hubei, China; 3 Swire Institute of Marine Science, School of Biological Sciences, University of Hong Kong, Pok Fu Lam, Hong Kong SAR, China

**Keywords:** *Aeromonas hydrophila*, antibiotic resistance, aquaculture, oxytetracycline, transcriptome, whole genome sequencing

## Abstract

**Introduction:**

The aquatic environment is an ideal ecosystem for the development of antimicrobial resistance (AMR) as it can be impacted by many sources of antibiotic residues.

**Methods:**

To better understand the mechanism for AMR associated with exposure to subtherapeutic concentrations of antibiotics, we monitored the genetic and transcriptional changes in *Aeromonas hydrophila* exposed to incremental doses of oxytetracycline (OTC), a commonly used antibiotic in aquaculture.

**Results:**

Our results showed that all three independent lineages rapidly developed OTC resistance, starting after 3 days of exposure, with consistent adaptation patterns across replicates. Whole-genome sequencing identified recurrent point mutations, particularly in genes *AHA_ 2,785*, *AHA_2910 (aheB)*, and *AHA_0308 (rpsJ)*. Transcriptomic analysis identified over 1,000 differentially expressed genes across bacterial replicates exposed to 100 ppm OTC, and further revealed coordinated changes in pathways associated with efflux pumps, outer membrane proteins, and ribosomal proteins.

**Discussion:**

These combined genetic and transcriptional changes likely drove the progressive development of resistance during incremental antibiotic exposure. Our findings support the notion that AMR can arise in aquatic bacteria through coordinated regulation of several intrinsic mechanisms and highlight potential risks during in-feed antibiotic usage in aquaculture.

## 1 Introduction

The rise of antimicrobial resistance (AMR), largely driven by overuse and inappropriate use of antibiotics, poses a significant challenge to human and veterinary medicine. Prolonged exposure to low-dose antibiotics, once common in food animal production, has been banned in many countries due to its role in promoting resistance (Wistrand-Yuen et al., 2018; Stanton et al., 2020). In aquaculture, however, animals can still be inadvertently exposed to subtherapeutic antibiotic levels during metaphylactic feed treatments (Price et al., 2019). This happens for a few reasons, but poor dosing and uneven feeding within the animal population are particularly problematic during treatments. In addition, aquatic environments are exposed to low levels of antibiotics from multiple non-aquaculture sources including agriculture runoff and wastewater discharge (Li et al., 2020; Yi et al., 2025). These factors together create conditions that favor the selection and amplification of AMR within microbial communities, including major fish pathogens such as *Aeromonas* spp., *Edwardsiella* spp., *Streptococcus* spp., and *Vibriosis* spp. (Taylor et al., 2011; Adenaya et al., 2023).

Among these, *Aeromonas hydrophila* is a prominent fish pathogen affecting a wide range of fish, including economically important species such as carp (*Cyprinus carpio*), channel catfish (*Ictalurus punctatus*), and striped bass (*Morone saxatilis*) (Ramesh and Souissi, 2018). This pathogen has also been reported in humans to cause gastroenteritis and wound infections via foodborne and nosocomial transmission (Pessoa et al., 2022; Yuwono et al., 2023; Jeamsripong et al., 2025). The pathogenicity of *A. hydrophila* is attributed to a diverse array of virulence factors, including hemolysin, aerolysin, enterotoxin and the ability to form biofilms (Semwal et al., 2023; Ashikur Rahman et al., 2024). In aquaculture, it is a major cause of motile Aeromonas septicemia (MAS). Fish infected with *A. hydrophila* commonly are reported to have hemorrhaging, abdominal distension, skin ulcers, and exophthalmia (Shirajum Monir et al., 2020). Mortality can be high particularly if there are other co-factors (i.e., water quality issues and or co-infections).

To control bacterial infections, antibiotics—particularly oxytetracycline (OTC)—have been widely used. *Aeromonas hydrophila* has been shown to develop resistance to antibiotics through multiple mechanisms (Ko et al., 1998; Li et al., 2021; Semwal et al., 2023). Tetracycline resistance in *A. hydrophila* is primarily mediated through three well-characterized mechanisms: active efflux pumps that reduce intracellular drug accumulation (e.g., t*etE*), ribosomal protection proteins that prevent tetracycline binding to the ribosomal subunit (e.g., *tetM*), and, less commonly, enzymatic inactivation of the antibiotic. These resistance determinants are frequently regulated by transcriptional repressors such as *tetR* and are often located on mobile genetic elements, facilitating horizontal gene transfer across bacterial populations (Dönhöfer, Franckenberg et al., 2012; Skwor et al., 2014; Suzuki et al., 2015; Jeamsripong et al., 2025). Consequently, the rise of AMR in *A. hydrophila* threatens both aquaculture sustainability and public health.

Sub-inhibitory concentrations of OTC are frequently detected in aquatic environments (Karmakar et al., 2024), and may also increase AMR selection. Despite this risk, the mechanisms by which exposure to subtherapeutic OTC drives resistance in *A. hydrophila* and whether such adaptations impose fitness costs are not well understood. In this study, we evaluated the molecular changes associated with the development of OTC resistance in *A. hydrophila.* Using a novel, stepwise exposure to increasing OTC concentrations, we studied the potential genetic mechanisms underpinning *A. hydrophila* resistance against OTC and determined whether the resulting phenotypic resistance conferred a fitness advantage in an OTC-free environment.

## 2 Materials and methods

*Aeromonas hydrophila* was selected for this study as it represents a common environmental fish pathogen (Shabana et al., 2025), and OTC was chosen because it is one of the most extensively used antibiotics in aquaculture globally (Schar et al., 2020) and it is frequently detected at low concentrations in aquatic environments (Yi et al., 2025). To determine the impact of subtherapeutic exposure to OTC on phenotypic resistance and the mechanisms for acquired resistance to this product, we exposed an isolate of *A. hydrophila*, which was phenotypically sensitive to OTC, and which did not possess any known *tet* genes, to incremental concentrations of this antibiotic (V6322, InvivoChem®) starting from 5 ppm up to 100 ppm. We evaluated the bacteria’s resistance to 100 ppm OTC once they grew on the different incremental subtherapeutic OTC concentrations, as this is the therapeutic dose used for many salmonid fish pathogens (Price et al., 2019). We also measured gene expression and examined changes in the bacteria’s genome profile as it progressed through the different concentration gradients of OTC. Lastly, we assessed the bacteria’s fitness in an antibiotic-free environment as they were selected for different concentrations of OTC.

### 2.1 Bacterial strain

*Aeromonas hydrophila* (ATCC 7966, Culti-Loops, R4601020, Thermo Fisher, USA) was amplified on tryptone soy agar (TSA) (CM0131, OXOID, UK) at 37 °C overnight. A single colony of *A. hydrophila* was then collected and cultured on a shaker plate in tryptone soy broth (TSB) (CM0129, OXOID, UK) at 250 rpm at 37 °C overnight. Bacterial concentration was then adjusted to 8 × 10^7^ cfu/mL (equivalent to OD600 of 1.6–1.7) using a photometer DEN-600 (BioSan, Latvia).

### 2.2 Antibiotic-resistance gradient plates

A two-step gradient plate system was used in this study because it created a spatially structured environment which enabled bacterial migration and antibiotic adaptation. In addition, our system permitted us to directly visualize bacterial movement across the antibiotic gradient. We built the two-step gradient plates based on the premise of the MEGA-plate model described in Baym et al. (2016). In brief, a stock solution of OTC (20,000 ppm) was made by dissolving 40 mg of OTC into 2 mL of DMSO (20,688, Thermo Fisher, USA). Different amounts of this stock solution were added to TSA after the agar had cooled to 50 °C to make the following concentrations of OTC: 5 ppm, 20 ppm, 50 ppm, 100 ppm and 200 ppm. We added DMSO (same concentration as in the 5 ppm OTC solution) to our 0 ppm media to control for the effect of DMSO on *A. hydrophila*. To create plates with different concentrations of OTC, we placed a small sterile petri dish (height = 1.3 cm, diameter = 5 cm) in the center of a larger petri dish (height = 2.4 cm, diameter = 14 cm) (BKMAN company of China). We then added 30 mL of the lower concentration of OTC to the inner small plate, and 190 mL of the next highest concentration of OTC TSA agar to the outer petri dish. This created antibiotic-resistant gradient plates with two incremental concentrations of OTC: 0–5 ppm (G5), 5–20 ppm (G20), 20–50 ppm (G50), 50–100 ppm (G100), and 100–200 ppm (G200) (Supplementary Figure 1). These concentrations of OTC were used as they theoretically could be found in medicated feeds during metaphylactic treatments in aquaculture (Price et al., 2019).

After the antibiotic agar (basal layer) solidified, 31 mL of a semi-liquid 0.5% TSA was poured to create a “swimming layer” over the entire plate, allowing bacteria to cross the inner petri dish wall to the outer petri dish, which had the higher OTC concentration. To suppress fungal contamination in the semi-liquid layer, 100 μg/mL Ketoconazole (R41400, MedChemExpress, USA) was added to the media before it was poured on the plates.

High-performance liquid chromatography with diode-array detection (HPLC-DAD) was used to assess the concentration of OTC in the semi-liquid layer of antibiotic-resistant gradient plates over time. In brief, we collected 2 mL of semi-liquid agar from either the center of a small petri dish with the lower OTC concentration, as well as the middle area of the large petri dish with the higher OTC concentration, at day 1 and day 4 in G5, G20, G50, and G100 (Supplementary Figure 1) from 3 replicate plates. Samples for HPLC-DAD in the plates with 100 ppm in the center base layer and 200 ppm in the outer base layer were collected on day 1 and day 3. Samples were analyzed at the Hong Kong Baptist University (HKBU) Chemistry laboratory for analysis.

### 2.3 Antibiotic resistance training procedure

To initiate antibiotic resistance training, we first inoculated 40 μL of our initial strain of *A. hydrophila* (R0) in the center of the swimming layer of the G5 plate. This was done in triplicate. All plates were cultured at 30 °C and examined daily for bacterial growth. If the bacteria did not cross into the outer ring of the training plates by day 3, we then collected and transferred 2 mL of bacteria from the edges of where the bacteria had migrated to the center of a new plate with the same concentration as OTC. The timing of the transfer was based on the HPLC-DAD results of the training plates, as we observed a reduction in the concentration of OTC and horizontal diffusion of the OTC over time (Supplementary Table1).

Once the bacteria had colonized half of the surface area of the outer plate with the higher OTC concentration, 2 mL of bacteria was collected from the leading edge of the bacterial colony and transferred to the center of the swimming layer of the next higher gradient plate (Supplementary Figure 1). Before doing this transfer, 2 mL of the swimming layer was removed from the center of the new plate to maintain the same volume of semi-liquid media. Meanwhile, we also sampled bacteria to assess their fitness and resistance to OTC (see methods below). Further, we collected 500 μL of bacteria and placed them in 500 μL of 30% glycerol, which was stored at −80 °C for genetic analysis (see methods below), including periodic 16S rRNA sequencing to confirm *A. hydrophilia* throughout our resistance training.

The entire process was repeated until the bacteria reached our last gradient plate (G200) with the inner base ring layer having a concentration of 100 ppm of OTC and the outer base layer ring containing 200 ppm of OTC. The swimming layer where the bacteria were growing was always less than the base layer (Supplementary Table 1), but in the case of the 200 ppm base layer the swimming layer was determined to have 151 ppm of OTC after 24 h, which was over the recommended therapeutic level for many bacterial pathogens (Price et al., 2019).

### 2.4 Assessing phenotypic resistance to OTC and bacterial fitness in a non-antibiotic environment

Each time we transferred bacteria to the next gradient training plates, we also tested its resistance to OTC in TSB and on TSA media. To do this we first amplified 300 μL of bacteria from different OTC training plate concentrations in 3 mL of TSB with the same concentration of OTC. Bacterial suspensions were cultured on a shaker platform at 250 rpm and 30 °C until they attained a concentration of 6 × 10^7^ cfu/mL (equivalent to OD600 of 1.0–1.2). We then added the inoculated TSB to both TSA and TSB with and without high concentration OTC (see below). The same culture procedure was used on the original *A. hydrophila* (R0) to compare fitness between the original bacteria and the isolates exposed to different concentrations of OTC. Each isolate was tested in duplicate.

#### 2.4.1 Test plates

The resistance test plates had two layers of TSA media. The swimming layer was identical to the swimming layer described for the training plates. The base layer of the negative test plates used to assess fitness in an antibiotic-free environment contained 220 mL of 2% TSA agar without OTC (used to assess the viability of the isolate). The base layer for the positive test plates was created with 2% TSA agar and contained 100 ppm or 200 ppm OTC (Supplementary Figure 2). To determine the OTC concentration in the swimming layer of the test plates over time, 2 mL of semi-liquid agar was collected at the center of the plate at day1, 4, and 5 and submitted to the HKBU laboratory for HPLC-DAD analysis (Supplementary Table 1).

The TSA plates were cultured at 30 °C for up to 5 days. An isolate was considered sensitive to OTC if it did not grow on the OTC test plates (100 ppm and 200 ppm) within 5 days. If the bacteria grew on the plates with OTC, it was considered resistant. The plates without OTC were used as internal controls to ensure the isolates were viable.

#### 2.4.2 Test broth

Bacterial resistance and fitness were also assessed using broth culture. Each of the bacterial suspension (1%) was added to TSB containing 113 ppm OTC (based on the HPLC-DAD results). The bacterial growth in this medium was periodically monitored using a spectrophotometer over a four-day period, specifically at time points 0, 2, 4, 6, and 8 h on each day (day 1, 2, 3, and 4). Based on the growth curve, we determined the growth log phase for this experiment to be on day 4. We compared the OD readings at day 4 for the different isolates (R0, R5, R20, R50, R100, R200) using a linear regression and Box-Cox transformation to stabilize the variance and meet the normality assumption.

To evaluate the fitness of the bacteria in an antibiotic-free environment, the same methods were employed with the exception that the TSB did not have OTC and bacterial growth was only monitored for one day at 0, 2, 4, 6, and 8 h. Each bacterial isolate was tested in duplicate. At the end of the designated time points, 100 μL of the bacteria were spread onto TSA plates without antibiotics. These plates were then incubated at 30 °C overnight to determine the viability of the bacteria.

The growth curves (i.e., OD levels) of the different bacterial isolates were compared at 4 h post-inoculation, as this was the peak log growth phase based on our data. We used a linear regression model with robust standard errors to compare OD readings of the bacteria selected for survival in different concentrations of OTC in TSB without antibiotics. Robust standard errors were used because the model residuals did not meet the assumption of equal variance.

### 2.5 Genetic analysis

Preserved bacteria were revived in TSB at the same concentration of OTC as that present in the semi-liquid layer of the training plate from which they originated. The specific concentration of OTC used for revival was determined based on the HPLC-DAD results. Bacterial suspensions were cultured on a shaker platform at 250 rpm and 30 °C until the concentrations reached approximately 10^9^ cfu/mL. The revived bacteria were subsequently used for DNA and RNA for genetic analysis. The DNA and RNA were extracted using TaKaRa MiniBEST Universal Genomic DNA Extraction Kit Ver.5.0 (TaKaRa Bio Inc., Japan) and HiPure Bacterial RNA Kit (R4181, Magen Biotech, China), respectively, as per manual instructions. Further, the DNA and RNA quality and quantity were determined by NanoDrop™ One/OneC Microvolume UV–Vis Spectrophotometer (Thermo Fisher, USA). Samples with concentrations of DNA and RNA greater than 10 ng/μL and 50 ng/μL, respectively, were submitted to Novogene (China) for whole-genome sequencing and RNA-seq.

### 2.6 Whole genome sequencing and analysis

#### 2.6.1 Library construction and sequencing

A total of 1 μg of DNA per sample was used as input material for DNA sample preparation. Sequencing libraries were generated using NEBNext® UltraTM DNA Library Prep Kit for Illumina (NEB, USA) following the manufacturer’s recommendations, and index codes were added to attribute sequences to each sample. Briefly, the DNA sample was fragmented by sonication to a size of 350 bp, then the DNA fragments were end-polished, A-tailed, and ligated with the full-length adaptor for Illumina sequencing with further PCR amplification. Subsequently, PCR products were purified (AMPure XP system) and libraries were analyzed for size distribution by Agilent2100 Bioanalyzer and quantified using real-time PCR. Sequencing then was performed on the Illumina NovaSeq PE150 platform (Beijing Novogene Bioinformatics Technology Co., Ltd.)

#### 2.6.2 Sequence read processing

Raw data (raw reads) were first processed through fastp software to obtain a final set of high-quality reads. Clean reads were obtained by removing reads containing adapters, reads containing ploy-N, and low-quality reads from raw data. To identify genetic variants in our samples, we mapped the reads to the designated reference genome (*Aeromonas hydrophila* subsp. *hydrophila* ATCC 7966^1^) using BWA software with the parameters mem -t 4 -k 32 -M -R. and the parameter depth set to -d 200,000 (V0.7.8, Officialdocumentation:^2^ manual:^3^, counting the coverage of the reference sequence to the reads SAMTOOLS software V0.1.18^4^) (McKenna et al., 2010; Danecek et al., 2021). This reference strain was used because it was the strain we purchased for this study (R0).

We focused on comparing the non-synonymous mutations resulting from single nucleotide polymorphisms (SNPs), insertion and deletion mutations (InDel), and alterations resulting in structural variations (SV) in the coding sequence (CDS) regions of genes, particularly genes that have been associated with AMR in the past.

#### 2.6.3 SNP (single nucleotide polymorphism)/INDEL(insertion or deletion) calling

SAMTOOLS was used for detection of individual SNPs, and insertions and deletions of small fragments(<50 bp), as well as the analysis of the variation of the ratio of these SNPs/InDels in the functional regions of the genome. The parameters for this analysis using SAMTOOLS were as follows: mpileup -m 2 -F 0.002 -d 10,000 -u -L 10000 (Cingolani et al., 2012).

#### 2.6.4 SV (structural variation) calling

Based on the mapping results, Manta software (Illumina, manta-v1.6.0) was used to detect large fragments of insertion, deletion, inversion, ectopic and other structural variations (Chen et al., 2016).

#### 2.6.5 CNV(copy number variation) calling

CNVs were detected using CNVnator (v0.4.1) (Abyzov et al., 2011) to acquire potential deletion and duplication (parameter: - call 100 default setting).

#### 2.6.6 Analysis of known antimicrobial resistance genes

To investigate the presence of known antimicrobial resistance genes (ARGs) in the isolates, we conducted a homology-based search using Resistance Gene Identifier (RGI) on.

Comprehensive Antibiotic Resistance Database (CARD) (Alcock et al., 2020) as well as ResFinder (Bortolaia et al., 2020).

### 2.7 RNA-seq

#### 2.7.1 Library construction, sequencing and analysis

A total of three independent biological replicates of R0, R5, R20, R50, R100, and R200 were used for RNA sequencing in Novogene Co. Ltd. (Hong Kong Subsidiary, China). Quantified libraries were sequenced on Illumina NovaSeq 6,000 (2 × 150 bp read length). The raw RNA-seq data have been deposited in NCBI’s Gene Expression Omnibus and are accessible through GEO Series accession number GSE290492. The genome of *A. hydrophila* ATCC 7966 from the National Center for Biotechnology Information (NCBI) GCA_000014805.1 was selected as the reference genome. We used Hisat2 to build the indices for the reference genome and the sample genomes. The GeneMarkS software^5^ was used to identify novel genes, operon and transcription start sites. The featureCount (Liao et al., 2014) command in the Subread software (V2.0.3) was used to count the reads numbers mapped to each gene.

#### 2.7.2 Gene functional annotation

Gene function annotation was performed using Prokka (Seemann, 2014) (v1.14.6), which employs a hierarchical annotation approach. The pipeline primarily relies on UniProtKB/Swiss-Prot for high-confidence annotations, followed by complementary annotation using the following databases: Pfam (protein domain identification via HMMER), COG/KOG (Clusters of Orthologous Groups for functional classification), and Prokka’s curated bacterial annotation database.

#### 2.7.3 DEG identification

The differentially expressed genes (DEGs) were selected based on the ratios between different treatment groups, which had greater than 1-fold change (FC) in the log base 2 gene expression transformation (i.e., |log2FC| > 1) with a *p* value less than 0.05 (Zhang et al., 2023). The DESeq2 (V1.46.0) (Love et al., 2014) R package was used to determine whether the differential expression between R0 and each isolate exposed to OTC was statistically significant. Furthermore, we visualized the statistically significant DEGs that were identical or unique across treatment groups using Venn diagrams^6^.

Additionally, we explored whether the DEGs that overlapped across OTC-exposed isolates were genes associated with efflux pumps, multidrug resistance, ribosomes, and outer membrane proteins, based on gene annotations.

#### 2.7.4 GO and KEGG pathway enrichment analysis

Functional enrichment analysis of differentially expressed genes (DEGs) was performed using the ClusterProfiler R package (v 4.14.4) (Yu et al., 2012) to identify over-represented Gene Ontology (GO) terms and Kyoto Encyclopedia of Genes and Genomes (KEGG) pathways (Kanehisa et al., 2025). For GO analysis, over-representation analysis (ORA) was conducted via the enricher function, using the hypergeometric test to assess enrichment of biological processes, molecular functions, and cellular components. KEGG pathway analysis was similarly performed using the KEGG enrichment function, with the same uncorrected threshold (*p* < 0.05) to identify metabolic and signaling pathways overrepresented among DEGs.

## 3 Results

Our WGS data confirmed that we had only *A. hydrophila* in our culture system during our study. The HPLC results of the training gradient and the test plates indicated that the concentration of OTC was always lower in the swimming layer than in the base layer (Supplementary Table 1), so we added a gradient plate and an additional test plate with a concentration of OTC in the base layer of 200 ppm OTC in order to achieve 100 ppm in the swimming layer. On day 1, the OTC concentration in the swimming layer of the larger petri dish in our gradient plates exceeded half of that in the base layer of the same dish. Similarly, the OTC concentration in the small petri dish swimming layer was also higher than half of the OTC concentration in the base layer (Supplementary Figure 1).

The OTC concentrations in the swimming layer of the training and test plates generally declined over time, with some samples having less than half of the concentration in the base layer by day 4 or 5. In the case of the G200 plates, the swimming layer had 113 ppm of OTC in the outer ring swimming layer, while the center of the swimming layer in the smaller inner plate had 79 ppm of OTC by day 3 (Supplementary Table 1). Replicate isolates of *A. hydrophila* never took more than 3 days to adapt to the next OTC concentration in the training plates.

### 3.1 Fitness and resistance of selected *A. hydrophila*

The results obtained from the test plates showed that all bacterial strains grew on plates without OTC after 24 h, suggesting they were all viable. It took 2 days before any growth occurred in the 5 ppm OTC concentration, suggesting the original isolate was not resistant to OTC. Further, no growth was observed in any of the positive PC100 and PC200 ppm OTC test plates for isolates collected from R0, R5, R20, and R50. There was partial colonization of the PC100 ppm positive test plate with the R100 isolates, but no growth in the 200 ppm test plate with these isolates. The R200 isolates were able to colonize both PC100 ppm and PC200 ppm test plates after 1 day, suggesting they were resistant to ~151 ppm of OTC (Supplementary Table 1).

**Figure 1:**
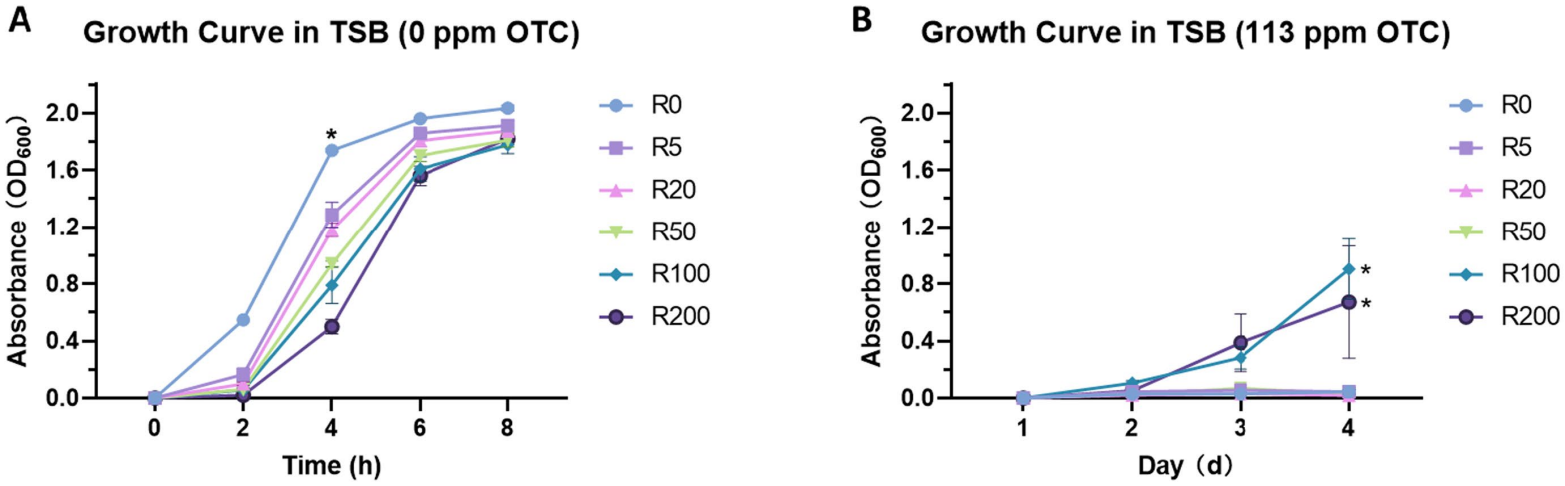
Fitness and resistance of isolates from training plates. **(A)** Bacterial growth in the OTC-free TSB. The level of resistance was significantly associated with the OD reading (*p* < 0.001). **(B)** Bacterial growth in TSB with 113 ppm of OTC over 4 days. Both R100 and R200 had significantly higher OD readings than R0 (*p* = 0.001 and *p* = 0.003, respectively). The mean of three biological replicates is shown, and error bars represent the standard error of the mean (SEM) (*n* = 3). *Indicates statistical significance.

The results obtained from the test broth without OTC suggested that the more resistant the isolate, the slower the growth (*p* < 0.001) after 4 h (Figure 1A). Furthermore, our linear regression model explained 67% of the variance in the OD readings. At 4 h post-inoculation, the level of resistance was inversely associated with the OD reading (*p* < 0.001).

**Figure 2:**
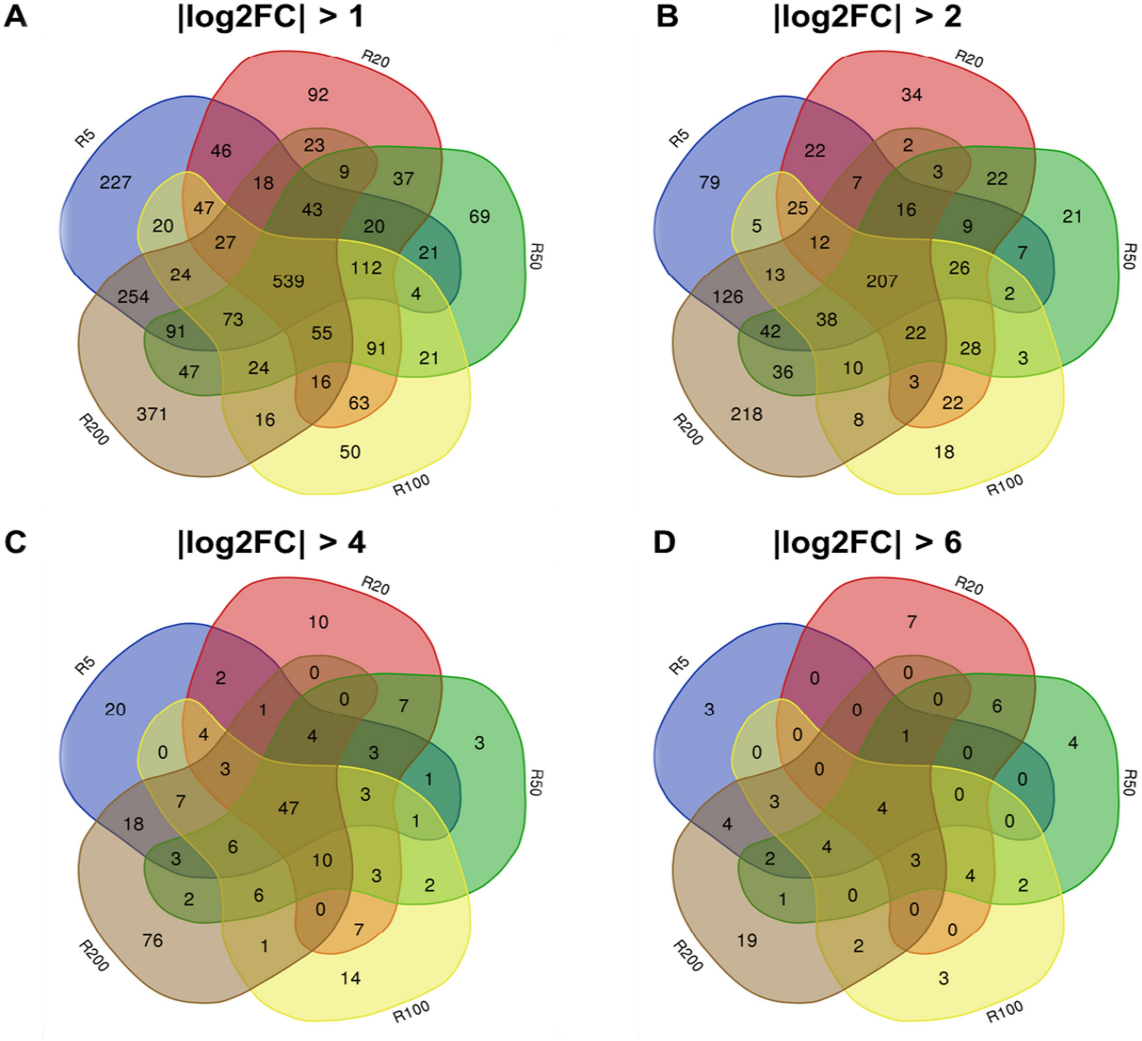
Venn diagrams of the DEGs of isolates from training plates. **(A)** Venn diagram of the DEGs with the criteria of |1og2FC| > 1 and *p* < 0.05; **(B)** Venn plot of the DEGs with the criteria of |log2FC| > 2 and *p* < 0.05; **(C)** Venn plot of the DEGs with the criteria of |log2FC| > 4 and *p* < 0.05;**(D)** Venn plot of the DEGs with the criteria of |log2FC| > 6 and *p* < 0.05.

For the growth analysis in TSB with 100 ppm OTC only the R100 and R200 isolates had any evidence of growth (Figure 1B). One of the three R200 isolates could not be revived. The OD readings for isolates selected to grow in this concentration of OTC were significantly different from those of the R0 group (*p* < 0.001 and *p* = 0.003). For the other low-dose-resistant isolates and for the R0, no increase in OD600 was observed in TSB with OTC (Figure 2B). Despite this, the bacteria collected from all the training plates with OTC (i.e., R5, R20, R50, R100, R200) were still viable on day 4, as evidenced by the presence of colonies on the TSA plates without OTC. In contrast, the bacteria from R0 exposed to TSB with 100 ppm of OTC for 4 continuous days was no longer able to grow on TSA at day 4.

### 3.2 Whole genome sequencing analysis

#### 3.2.1 Genome features of isolates

The whole genome sequences have been archived with NCBI (Bioproject # PRJNA1302019). All isolates mapped to the reference strain (*A. hydrophila* ATCC 7966) had alignment rates greater than 97.6% (Supplementary Table 2). Although the isolates were genetically similar, a few base-pair differences were found in the coding sequence (CDS) regions of different genes that may have contributed to the resistance patterns. The differences between each isolate and the reference genome are shown in Figure 2.

The whole genome sequencing revealed that the original strain, *A. hydrophila* ATCC 7966, contained multiple known antibiotic resistance genes (ARGs), a few of which may have an effect on antibiotic resistance (Table 1; Supplementary data sheet X1); however, R0 was never sensitive to 100 ppm of OTC on any of our test plates and it did not contain any *tet* genes specific for OTC resistance. Further, this isolate did not grow at 5 ppm for at least 2 days, suggesting it was initially sensitive to OTC.

**Table 1:** Identification of ARGs in the genome of *A. hydrophila* ATCC 7966 strain, according to CARD (Alcock et al., 2020) and ResFinder databases (Bortolaia et al., 2020).

Database	ARG	Gene id	ARGs family	Resistance mechanism	Drug class
CARD & ResFinder	imiS	AHA_3,712	CphA beta-lactamase	Antibiotic inactivation	Carbapenem
CARD	ArnT	AHA-0988	Pmr phosphoethanolamine transferase	Antibiotic target alteration	Peptide antibiotic
CARD	qacJ	AHA_1324	Small multidrug resistance (SMR) antibiotic efflux pump	Antibiotic efflux	Disinfecting agents and antiseptics
CARD	AcrB	AHA_2910	Resistance-nodulation-cell division (RND) antibiotic efflux pump	Antibiotic efflux	Fluoroquinolone antibiotic; tetracycline antibiotic
CARD & ResFinder	CepS	AHA_3135	CepS beta-lactamase	Antibiotic inactivation	Cephalosporin
CARD	rsmA	AHA_ 3,712	Resistance-nodulation-cell division (RND) antibiotic efflux pump	Antibiotic efflux	Fluoroquinolone antibiotic; diaminopyrimidine antibiotic; phenicol antibiotic
CARD & ResFinder	OXA-726/Amps	AHA_4258	OXA beta-lactamase; OXA-12-like beta-lactamase	Antibiotic inactivation	Penam
CARD	Tuf	AHA_4018	Elfamycin resistant EF-Tu	Antibiotic target alteration	Elfamycin antibiotic
ResFinder	Mcr-7.1	AHA_2670	MCR phosphoethanolamine transferase	Antibiotic target alteration	Peptide antibiotic

All of these genes were found in all of our isolates. The RGI quality assurance criteria were all above the acceptable reliability threshold.

There were 4 common mutations observed in all our isolates, including R0 (i.e., *AHA_4166*, *AHA_3698*, *AHA_2667*, and *AHA_3364*) (Figure 2). There was one gene mutation, which occurred in all isolates exposed to OTC. This gene was associated with an outer cell membrane protein (*AHA_2,785*). There was also one other gene mutation which was common to all isolates except R5-2 (*AHA_2910*). This gene was a known efflux pump ARG. There were several other gene mutations in our samples, but these were different between replicates and OTC exposure groups (Figure 2). Overall, there was a trend suggesting an increase in point mutations as the bacteria were exposed to more elevated concentrations of OTC, particularly in the second replicate series. The isolates that were selected to grow in the highest level of OTC all contained a mutation in the *AHA_0308* gene, which codes for the 30S ribosomal protein S10 that regulates translation, ribosomal structure and biogenesis.

### 3.3 RNA-seq library construction, sequencing and analysis

#### 3.3.1 Quality assessment and comparative analysis with the reference genome

The quality assessment of the isolates’ transcriptome sequencing is listed in Supplementary Table 3. The trimmed rates were above 95% (Q20) for all isolates. The RNA gene expression data has been deposited in Gene Expression Omnibus (GEO) (GSE290492).

#### 3.3.2 Analysis of DEGs

**Figure 3:**
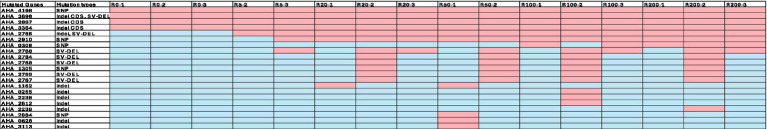
Table identifying gene mutations across different isolates exposed to different concentrations of OTC in our study.

All DEGs for each OTC exposure group are provided in a Supplementary data sheet X2. Among these, there were 920, 641, 714, 651, and 934 upregulated genes in R5, R20, R50, R100, and R200, respectively, compared with R0. While there were 646, 597, 542, 531, and 696 genes that were downregulated in R5, R20, R50, R100, and R200, respectively (Figures 3A–D). Furthermore, the Venn diagram showed that there were 539 overlapping DEGs across all treatments (R5, R20, R50, R100, and R200) (Figure 3A). The number of commonly overlapped and unique DEGs decreased as the |log_2_FC| cut-off increased across all treatments (Figure 3). When the |log_2_FC| cut-off was greater than 6, there were only 4 overlapping DEGs (*AHA_0907*, *AHA_2057*, *AHA_1248*, *AHA_2056*) across all isolates and 19 unique DEGs in R200 isolates (Figure 3D). The list of DEGs overlapping in the Venn diagram is provided in the Supplementary data sheet X4.

Among the 539 overlapping DEGs (|log_2_FC| > 1) common to all treatments, we found six genes (*AHA_1194*, *AHA_2910*, *AHA_2911 (acrA)*, *AHA_1713*, *AHA_2425*, *AHA_3960*) associated with the efflux pump system based on the gene annotation (Table 2). Five genes (*AHA_0354*, *AHA_0012*, *AHA_0853*, *AHA_3527*, *AHA_3528*), known to be associated with multidrug resistance, were also differentially regulated in all our OTC-exposed isolates (Table 2). Three of these were upregulated and 2 were downregulated (Table 2). There were also five overlapping DEGs (*AHA_3953*, *AHA_2909*, *AHA_1098*, *AHA_1280*, *AHA_3793*), which were associated with outer membrane proteins. All except one of these were up-regulated (Table 2). Several DEGs related to efflux pumps, multidrug resistance, and outer membrane proteins were also uniquely present and differentially expressed in R200. For example, gene *AHA_3053* which is associated with the efflux pumps and gene *AHA_0308*, which had a SNP and is associated with the small ribosomal subunit protein S10, were uniquely upregulated in R200. Gene *AHA_3507,* which is related to a multidrug transport system, was uniquely downregulated in R200. The outer membrane protein-related genes (*AHA_0897*, *AHA_4200*, *AHA_0074, AHA_1762*) were uniquely up-regulated in R200. Several genes associated with ribosomal proteins were also upregulated across all OTC-exposed isolates (Table 2).

**Table 2:** The DEGs potentially related to antimicrobial resistance based on gene annotation.

DEG categories	Log_2_FC	Gene id	Gene name	Protein names
Overlapping DEGs across all OTC exposure isolates	Up-regulation	*AHA_2910*	*aheB*	Efflux pump membrane transporter
		*AHA_2911*	*acrA*	Acriflavin resistance protein A (Membrane fusion protein)
		*AHA_1713*	*NA*	Bcr/CflA family efflux transporter
		*AHA_2425*	*NA*	ABC type drug efflux transporter, fused ATP binding and permease domains
		*AHA_3960*	*NA*	Cation efflux family protein
		*AHA_0354*	*NA*	Multidrug resistance protein B
		*AHA_0012*	*NA*	Multidrug resistance protein D
		*AHA_0853*	*mdtH*	Multidrug resistance protein MdtH
		*AHA_3953*	*NA*	Outer membrane protein W
		*AHA_2909*	*NA*	Outer membrane protein OprM
		*AHA_1098*	*NA*	Outer membrane protein beta-barrel domain-containing protein
		*AHA_1280*	*NA*	Major outer membrane protein OmpAII
		*AHA_0666*	*rpsP*	Small ribosomal subunit protein bS16 (30S ribosomal protein S16)
		*AHA_0308*	*rpsJ*	Small ribosomal subunit protein uS10
		*AHA_0322*	*rpsN*	Small ribosomal subunit protein uS14 (30S ribosomal protein S14)
		*AHA_0318*	*rpsQ*	Small ribosomal subunit protein uS17 (30S ribosomal protein S17)
		*AHA_0315*	*rpsC*	Small ribosomal subunit protein uS3 (30S ribosomal protein S3)
		*AHA_4020*	*rpsG*	Small ribosomal subunit protein uS7 (30S ribosomal protein S7)
		*AHA_3904*	*rpsI*	Small ribosomal subunit protein uS9 (30S ribosomal protein S9)
		*AHA_0332*	*rpsD*	Small ribosomal subunit protein uS4 (30S ribosomal protein S4)
		*AHA_0326*	*rpsE*	Small ribosomal subunit protein uS5 (30S ribosomal protein S5)
		*AHA_0323*	*rpsH*	Small ribosomal subunit protein uS8 (30S ribosomal protein S8)
		*AHA_0313*	*rpsS*	Small ribosomal subunit protein uS19 (30S ribosomal protein S19)
		*AHA_0334*	*rplQ*	Large ribosomal subunit protein bL17 (50S ribosomal protein L17)
		*AHA_0325*	*rplR*	Large ribosomal subunit protein uL18 (50S ribosomal protein L18)
		*AHA_0314*	*rplV*	Large ribosomal subunit protein uL22 (50S ribosomal protein L22)
		*AHA_4030*	*rplJ*	Large ribosomal subunit protein uL10 (50S ribosomal protein L10)
		*AHA_0328*	*rplO*	Large ribosomal subunit protein uL15 (50S ribosomal protein L15)
		*AHA_0321*	*rplE*	Large ribosomal subunit protein uL5 (50S ribosomal protein L5)
		*AHA_0310*	*rplD*	Large ribosomal subunit protein uL4 (50S ribosomal protein L4)
	
		*AHA_0309*	*rplC*	Large ribosomal subunit protein uL3 (50S ribosomal protein L3)
		*AHA_0327*	*rpmD*	Large ribosomal subunit protein uL30 (50S ribosomal protein L30)
		*AHA_0311*	*rplW*	Large ribosomal subunit protein uL23 (50S ribosomal protein L23)
		*AHA_0669*	*rplS*	Large ribosomal subunit protein bL19 (50S ribosomal protein L19)
		*AHA_0316*	*rplP*	Large ribosomal subunit protein uL16 (50S ribosomal protein L16)
		*AHA_3905*	*rplM*	Large ribosomal subunit protein uL13 (50S ribosomal protein L13)
		*AHA_0324*	*rplF*	Large ribosomal subunit protein uL6 (50S ribosomal protein L6)
		*AHA_4029*	*rplL*	Large ribosomal subunit protein bL12 (50S ribosomal protein L7/L12)
		*AHA_0317*	*rpmC*	Large ribosomal subunit protein uL29
		*AHA_0312*	*rplB*	Large ribosomal subunit protein uL2 (50S ribosomal protein L2)
		*AHA_4031*	*rplA*	Large ribosomal subunit protein uL1 (50S ribosomal protein L1)
		*AHA_0667*	*rimM*	Ribosome maturation factor RimM
*AHA_3157*	*ychF*	Ribosome-binding ATPase YchF

	Down-regulation	*AHA_3775*	*NA*	Ribosomal protein serine acetyltransferase
		*AHA_1557*	*NA*	Ribosomal S4P (Gammaproteobacterial)
		*AHA_1194*	*NA*	Cation (Na + −coupled) multidrug resistance efflux pump
		*AHA_3527*	*NA*	Multidrug resistance protein, SMR family
		*AHA_3528*	*NA*	Multidrug resistance protein, SMR family
*AHA_3793*		*NA*	Outer membrane protein P5
Unique DEGs in R200	Up-regulation	*AHA_3053*	*kefA*	Potassium efflux system KefA
		*AHA_0897*	*bamC*	Outer membrane protein assembly factor BamC
		*AHA_4200*	*NA*	Outer-membrane protein A
		*AHA_0074*	*btuB*	Vitamin B12 transporter BtuB (Cobalamin receptor) (Outer membrane cobalamin translocator)
		*AHA_1762*	*bamB*	Outer membrane protein assembly factor BamB
		*AHA_0318*	*rpsQ*	Small ribosomal subunit protein uS17 (30S ribosomal protein S17)
		*AHA_0313*	*rpsS*	Small ribosomal subunit protein uS19 (30S ribosomal protein S19)
		*AHA_0314*	*rplV*	Large ribosomal subunit protein uL22 (50S ribosomal protein L22)
		*AHA_0316*	*rplP*	Large ribosomal subunit protein uL16 (50S ribosomal protein L16)
		*AHA_0679*	*rpsT*	Small ribosomal subunit protein bS20 (30S ribosomal protein S20)
		*AHA_0931*	*rpmA*	Large ribosomal subunit protein bL27 (50S ribosomal protein L27)
		*AHA_4197*	*rpmE-2 rpmE*	Large ribosomal subunit protein bL31
		*AHA_3305*	*rimP*	Ribosome maturation factor RimP
		*AHA_3574*	*rlmJ*	Ribosomal RNA large subunit methyltransferase J (EC 2.1.1.266) (23S rRNA (adenine(2030)-N6)-methyltransferase) (23S rRNA m6A2030 methyltransferase)
		*AHA_1176*	*frr*	Ribosome-recycling factor (RRF) (Ribosome-releasing factor)
		*AHA_2790*	*NA*	Ribosome biogenesis GTPase A
	Down-regulation	*AHA_3507*	*NA*	ABC-type multidrug transport system, permease component

#### 3.3.3 GO annotation and KEGG pathway analysis of DEGs

**Figure 4:**
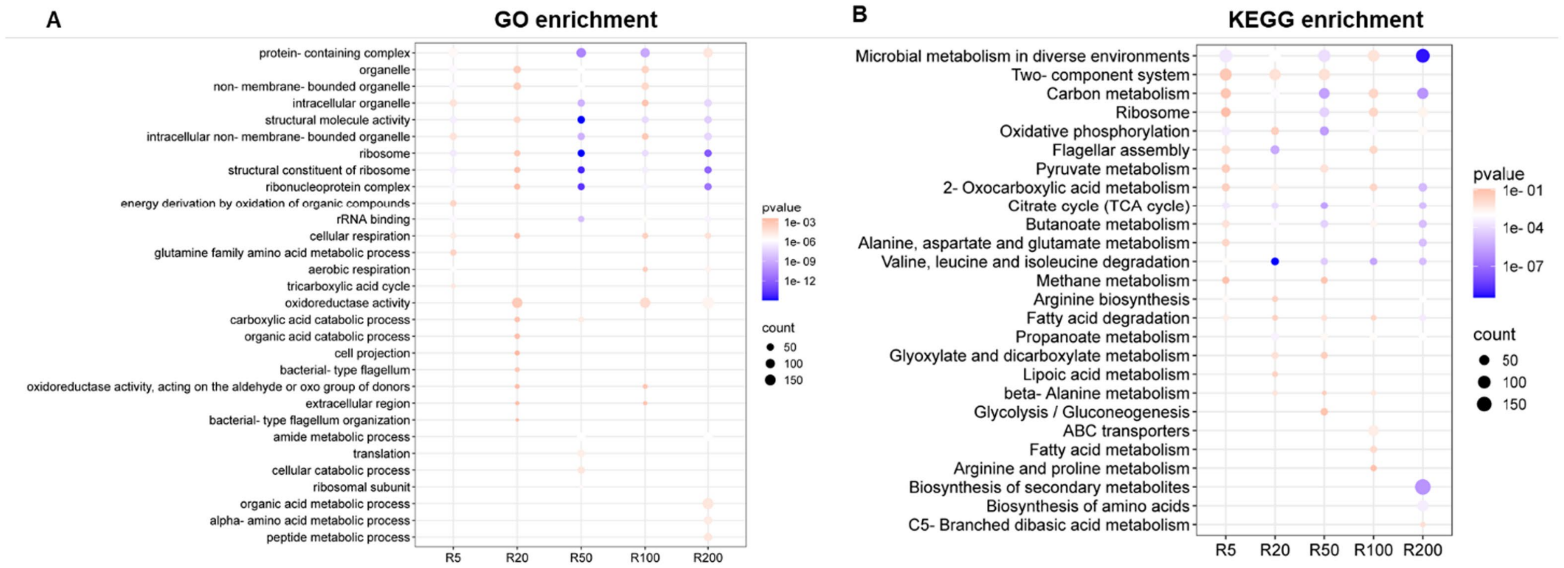
The top 15 GO enrichment **(A)** and KEGG pathways **(B)** (Kanehisa et al., 2025) across different treatment groups exposed to different concentrations of OTC.

The top 15 enriched GO terms and KEGG pathways using information on the DEGs in each OTC exposure group are shown in Figure 4. Among these, there were four overlapped GO terms across all exposure groups: ribonucleoprotein complex, ribosome, structural constituent of ribosome, and structural molecule activity. The GO terms protein-containing complex and intracellular non-membrane-bound organelle were enriched in all but isolates except R20. There were three unique GO terms present only in R200: organic acid metabolic process, alpha-amino acid metabolic process, and peptide metabolic process. The latter were driven predominantly by up-regulated DEGs.

For KEGG pathways, there were seven overlapped enriched pathways across all exposure groups: butanoate metabolism, carbon metabolism, citrate cycle (TCA cycle), fatty acid degradation, microbial metabolism in diverse environments, oxidative phosphorylation, and valine, leucine and isoleucine degradation. There were two pathways (ribosome and propanoate metabolism) that were consistently enriched in all isolates, but not in those exposed to 20 ppm or 5 ppm, respectively. Lastly, there were three enriched pathways uniquely in R200: biosynthesis of secondary metabolites, biosynthesis of amino acids, and C5-Branched dibasic acid metabolism.

## 4 Discussion

Bacteria develop antibiotic resistance through several well-documented genetically regulated mechanisms, including active drug export via efflux pumps, reduced antibiotic permeability through outer membrane modification, target protection or modification that prevents antibiotic binding, enzymatic inactivation of antibiotics, and target bypass via functional replacement of essential proteins (Darby et al., 2023). In our study, we observed evidence of several of these mechanisms during the evolution of OTC resistance in *A. hydrophila*. Following stepwise exposure, all three independent lineages rapidly developed resistance to therapeutic levels of OTC (i.e., ≥100 ppm) with highly consistent adaptive trajectories. The reproducibility of our findings across replicate isolates, together with the recurrence of specific mutations observed in WGS analysis, suggests a non-random process potentially shaped by antibiotic selective pressure. Consistent with this, transcriptome analysis revealed several DEGs associated with three classic intrinsic AMR mechanisms: efflux pumps, membrane-associated processes, and ribosomal function, which likely represent key drivers in the development of OTC resistance in *A. hydrophila*. Although some AMR-associated genes were identified in our parental *A. hydrophila* (R0) based on CARD (Alcock et al., 2020) and ResFinder databases (Table 1) (Bortolaia et al., 2020), none of the R0 isolates had *tet* genes, and all three R0 replicates were initially sensitive to OTC at 5 ppm, indicating that resistance is not solely determined by gene presence but also by gene regulation.

Efflux systems have been described to play a central role in the excretion of antibiotics from *A. hydrophila* (Hernould et al., 2008; Cruz et al., 2015). In particular, *AHA_2910* (*aheB*) encoding resistance-nodulation cell division (RND) efflux pump closely related to the *Escherichia coli* (*E.coli*) AcrB system (Ruiz and Levy, 2014), showed both consistent upregulation and recurrent mutation in our isolates. This suggests selection and functional adaptation. Its flanking genes *AHA_2909* and *AHA_2911* were also consistently upregulated but without detectable mutations, suggesting coordinated transcriptional activation. Both *AHA_2909* and *AHA_2911* encode membrane-associated proteins that are identified as multidrug efflux pumps in other bacteria (Hazel et al., 2019, Grosjean et al., 2021; Yu et al., 2021; Arrieta-Ortiz et al., 2023). More interestingly, these three genes shared high homology to the tripartite AheABC efflux pump previously characterized in *A. hydrophila* ATCC 7966, a system known to export multiple antibiotics (Hernould et al., 2008). In addition, isolates which grew in 100 ppm OTC (R200) showed upregulation of *kefA* (*AHA_3035*), a potassium efflux gene associated with acriflavine resistance components encoded by *AHA_2911*, *AHA_2910*, and *acrR* (Hadchity et al., 2021), suggesting several efflux pump proteins function together (Ma et al., 2016). Several other efflux-related genes (i.e., *AHA_1713*, *AHA_2425*, *AHA_3960*) were also upregulated across OTC-exposed isolates. Collectively, the activation of multiple efflux pumps over time likely contributed to the incremental resistance to OTC.

In addition to efflux pumps, changes with outer membrane-associated proteins were also a prominent finding in our study. For example, gene *AHA_2,785* was associated with an outer membrane protein that can negatively regulate the entry of OTC into bacteria (Zhang et al., 2022). Interestingly, this gene carried a recurrent point mutation across all OTC-exposed lineages; however, this mutation in the isolates exposure to lower OTC concentrations did not confer high-level resistance nor consistently alter expression (Supplementary data sheet X2), suggesting a potential modulatory rather than determinative role. Transcriptomic data further indicated broad regulation of outer membrane components (Table 2). For instance, *OmpAII* (*AHA_1280*), a known membrane target of antimicrobial compounds (Srinivas et al., 2010; Luther et al., 2019), was up-regulated in all isolates exposed to OTC (Supplementary data sheet X4). In highly resistant isolates (R200), there was an increased expression of *lamb* (*AHA_1166*), which is associated with multidrug resistance in *E.coli* (Bianchi et al., 2023). Additionally, *bamB* (*AHA_1762*) and *bamC* (*AHA_0897*), components of the beta-barrel assembly machinery (BAM) complex responsible for proper folding and insertion of outer membrane protein (Hayashi et al., 2024), were up-regulated in R200 isolates (Table 2). Given that the BAM complex is highly conserved among Gram-negative bacteria and essential for outer membrane function (Kim and Paetzel, 2011), including antibiotic expulsion (Daury et al., 2016, Xu et al., 2023), its activation may reflect adaptive membrane remodeling, thereby facilitating survival under antibiotic stress.

Ribosomal pathways, the primary target of OTC, were also significantly affected. GO and KEGG analyses showed enrichment of translation-related pathways, with widespread upregulation of ribosomal protein genes across OTC-exposed isolates (Figure 4, Table 2 and Supplementary data sheet X4). In particular, the 30S ribosome subunit proteins S17 and S19, encoded by *rpsQ (AHA_0318)* and *rpsS (AHA_0313)*, respectively, were highly up-regulated and are targets for OTC (Chukwudi, 2016). Overexpression of genes involved in this structure may have contributed to the resistance to OTC. In addition, in isolates R100 and R200 a recurrent mutation in *AHA_0308 (rpsJ)*, encoding the S10 protein (Figure 2), was detected alongside its consistent upregulation (Table 2 and Supplementary data sheet X4). These combined changes may alter the antibiotic target site or increase ribosomal capacity, contributing to resistance. Confirmation of the causal pathways for resistance mechanisms hypothesized in this study require further functional validation studies.

Beyond these primary mechanisms, GO and KEGG analyses suggested broad metabolic reprogramming. Genes involved in energy production and metabolic activity, such as aerobic respiration, oxidoreductase activity, sulfur metabolism, fatty acid degradation, and various catabolic processes, were also enriched (Figure 4). Many of the involved genes in these enrichments were significantly down-regulated across the OTC-exposed isolates (Supplementary data sheet X2 and X3). Similar metabolic suppression has been documented in resistant aquatic bacterial pathogens (Lin et al., 2015; Yu et al., 2021; Liu et al., 2023) and may reflect an energy conservation strategy under stress. Consistent with this, OTC-resistant isolates showed reduced growth in antibiotic-free media, indicating a fitness cost (Figure 1A) (Lin et al., 2015). This trade-off suggests that selection for resistance to OTC may be partially reversible in the absence of selective pressure (Motta et al., 2015), although its stability in aquaculture settings remains unclear and warrants further investigation.

Overall, our results showed that OTC resistance in *A. hydrophila* may have occurred from coordinated genetic and transcriptional adaptation. Notably, resistance emerged rapidly under laboratory conditions where bacteria were exposed to subtherapeutic levels of OTC, underscoring the challenge of preventing AMR. The consistent involvement of non-specific pathways, including efflux pumps and outer membrane regulation, also suggests potential cross-resistance to other antibiotics, highlighting the need for improved dosing strategies and antibiotic stewardship. Future studies should examine whether similar evolutionary dynamics occur under field environments, where exposed to subtherapeutic antibiotic levels due to variable in-feed dosing, sewage discharge, agricultural runoff, and medication use occurs.

## 5 Conclusion

The process of developing OTC resistance likely arose through a complex process involving both limited genetic mutations and extensive transcriptional changes. Despite relatively few SNPs and InDels, their recurrence across independent replicates suggested a non-random selection process. Together with consistent transcriptional changes, these adaptations generated a stable resistant phenotype despite slight lineage-specific variation in mutations and gene expression. Resistance likely emerged through multiple adaptive routes, with consistent involvement of key pathways including efflux pumps, outer membrane regulation, and ribosomal function, supporting a multi-tiered model rather than a single deterministic mechanism (Darby et al., 2023). The predominance of transcriptional changes further suggests that resistance may be partially reversible in the absence of antibiotic pressure, consistent with observed fitness costs under antibiotic-free conditions, although its stability in natural environments remains uncertain. Overall, the diversity of underlying mechanisms highlights the challenge of genetic monitoring, as no single pathway or definitive marker governs OTC resistance.

## Data Availability

The whole genome sequences have been archived with NCBI (Bioproject # PRJNA1302019). The RNA gene expression data has been deposited in Gene Expression Omnibus (GEO) (GSE290492).

## Footnotes

1
https://www.ncbi.nlm.nih.gov/nuccore/NC_008570.1?report=fasta&to=4744448

2
http://bio-bwa.sourceforge.net/

3
http://biobwa.sourceforge.net/bwa.shtml

4
http://www.htslib.org/

5
http://topaz.gatech.edu

6
http://bioinformatics.psb.ugent.be/webtools/Venn/
