# Supplementary material for: Antibiotic resistance in Aeromonas hydrophila associated with exposure to subtherapeutic levels of oxytetracycline
Source: Front Microbiol. 2026 May 8;17:1794341. doi: 10.3389/fmicb.2026.1794341 (PMC13194095; doi:10.3389/fmicb.2026.1794341)
Supplement: Supplementary file 2 [file Table_2.DOCX]

**Supplementary Table 1.** The averaged OTC concentration (ppm) in the swimming layer. Day 1 was 24 hours after the OTC was added to the base layer media.

| **Plate ID** | **Base layer OTC concentration** | **Day 1**  **Swimming layer** | **Day 4***  **Swimming layer** | **Day 5**  **Swimming layer** |
| --- | --- | --- | --- | --- |
| G5-Xa (inner ring) | 0 | <3 | <3 | NA |
| G5-Xb (outer ring) | 5 | <3 | 4 | NA |
| G20-Xa | 5 | 4 | 6 | NA |
| G20-Xb | 20 | 12 | 10 | NA |
| G50-Xa | 20 | 15 | 13 | NA |
| G50-Xb | 50 | 33 | 25 | NA |
| G100-Xa | 50 | 33 | 26 | NA |
| G100-Xb | 100 | 66 | 42 | NA |
| G200-Xa | 100 | 90 | 79* | NA |
| G200-Xb | 200 | 154 | 113* | NA |
| PC 100ppm | 100 | 63 | 54 | 48 |
| PC 200ppm | 200 | 151 | 113 | 99 |

NA: Not applicable.

## *OTC concentration for G200 was from Day3.

**Supplemental Table 2**. Genetic comparison between isolates and reference *A. hydrophila* ATCC 7966. All isolates were done in triplicate.

| **Isolates** | **Mapped rate (%)** |
| --- | --- |
| Replicate1 |  |
| R0-1 | 99.85 |
| R20-1 | 98.02 |
| R50-1 | 98.48 |
| R100-1 | 96.65 |
| R200-1 | 99.49 |
| Replicate2 |  |
| R0-2 | 99.84 |
| R5-2 | 98.44 |
| R20-2 | 97.61 |
| R50-2 | 98.24 |
| R100-2 | 96.8 |
| R200-2 | 99.43 |
| Repilicate3 |  |
| R0-3 | 99.82 |
| R5-3 | 98.51 |
| R20-3 | 98.24 |
| R100-3 | 99.27 |
| R200-3 | 99.3 |

**Supplementary Table 3.** The RNA transcriptome sequencing quality assessment parameters for all isolates.

| **Sample name** | **Raw reads** | **Clean reads** | **Raw bases** | **Clean bases** | **Error rate (%)** | **Q20(%)** | **Q30(%)** | **GC content (%)** |
| --- | --- | --- | --- | --- | --- | --- | --- | --- |
| R0_1 | 9,553,768 | 9,209,310 | 1.4G | 1.4G | 0.02 | 98.2 | 95.15 | 57.29 |
| R0_2 | 10,375,758 | 10,021,694 | 1.6G | 1.5G | 0.02 | 97.85 | 94.57 | 56.23 |
| R0_3 | 9,352,340 | 9,005,696 | 1.4G | 1.4G | 0.02 | 98.08 | 94.93 | 57.16 |
| R5_1 | 8,819,654 | 8,658,418 | 1.3G | 1.3G | 0.02 | 98.59 | 95.51 | 58.75 |
| R5_2 | 8,866,104 | 8,675,832 | 1.3G | 1.3G | 0.02 | 98.58 | 95.49 | 57.97 |
| R5_3 | 9,756,098 | 9,569,422 | 1.5G | 1.4G | 0.02 | 98.5 | 95.33 | 57.37 |
| R20_1 | 9,796,072 | 9,584,570 | 1.5G | 1.4G | 0.02 | 98.41 | 95.09 | 58.15 |
| R20_2 | 9,280,050 | 8,738,642 | 1.4G | 1.3G | 0.02 | 98.52 | 95.43 | 57.74 |
| R20_3 | 11,952,338 | 11,814,928 | 1.8G | 1.8G | 0.02 | 98.5 | 95.38 | 58.36 |
| R50_1 | 9,744,130 | 9,611,506 | 1.5G | 1.4G | 0.02 | 98.26 | 94.73 | 57.75 |
| R50_2 | 9,426,496 | 9,228,648 | 1.4G | 1.4G | 0.02 | 98.3 | 94.91 | 56.81 |
| R50_3 | 7,142,768 | 7,007,254 | 1.1G | 1.1G | 0.02 | 98.51 | 95.33 | 55.19 |
| R100_1 | 9,537,488 | 9,372,184 | 1.4G | 1.4G | 0.02 | 98.17 | 94.55 | 57.83 |
| R100_2 | 9,482,958 | 9,312,582 | 1.4G | 1.4G | 0.02 | 98.43 | 95.16 | 57.07 |
| R100_3 | 8,943,398 | 8,763,160 | 1.3G | 1.3G | 0.03 | 97.76 | 93.86 | 54.76 |
| R200_1 | 10,036,440 | 9,629,036 | 1.5G | 1.4G | 0.02 | 98.37 | 95.34 | 55.75 |
| R200_2 | 8,981,722 | 8,714,114 | 1.3G | 1.3G | 0.02 | 98.44 | 95.54 | 55.37 |
| R200_3 | 10,815,184 | 10,626,594 | 1.6G | 1.6G | 0.02 | 98.01 | 94.38 | 57.97 |

**Supplementary Table 4.** Number of unique DEGs in each treatment or overlapped DGEs across all treatments**.**

|  | **Treatment** | **\|Log2FC\| > 1** | **\|Log2FC\| > 2** | **\|Log2FC\| > 4** | **\|Log2FC\| > 6** |
| --- | --- | --- | --- | --- | --- |
| All DEGs | R5 | 227 | 79 | 20 | 3 |
|  | R20 | 92 | 34 | 10 | 7 |
|  | R50 | 69 | 21 | 3 | 4 |
|  | R100 | 50 | 18 | 14 | 3 |
|  | R200 | 371 | 218 | 76 | 19 |
|  | overlapped | 539 | 206 | 47 | 4 |
| Up-regulated DEGs | R5 | 174 | 66 | 17 | 1 |
|  | R20 | 60 | 15 | 5 | 7 |
|  | R50 | 30 | 13 | 3 | 4 |
|  | R100 | 38 | 11 | 9 | 3 |
|  | R200 | 196 | 117 | 35 | 3 |
|  | overlapped | 296 | 126 | 30 | 1 |
| Down-regulated DEGs | R5 | 63 | 14 | 3 | 2 |
|  | R20 | 55 | 20 | 5 | 0 |
|  | R50 | 41 | 8 | 0 | 0 |
|  | R100 | 19 | 8 | 5 | 0 |
|  | R200 | 206 | 104 | 41 | 16 |
|  | overlapped | 243 | 80 | 17 | 3 |


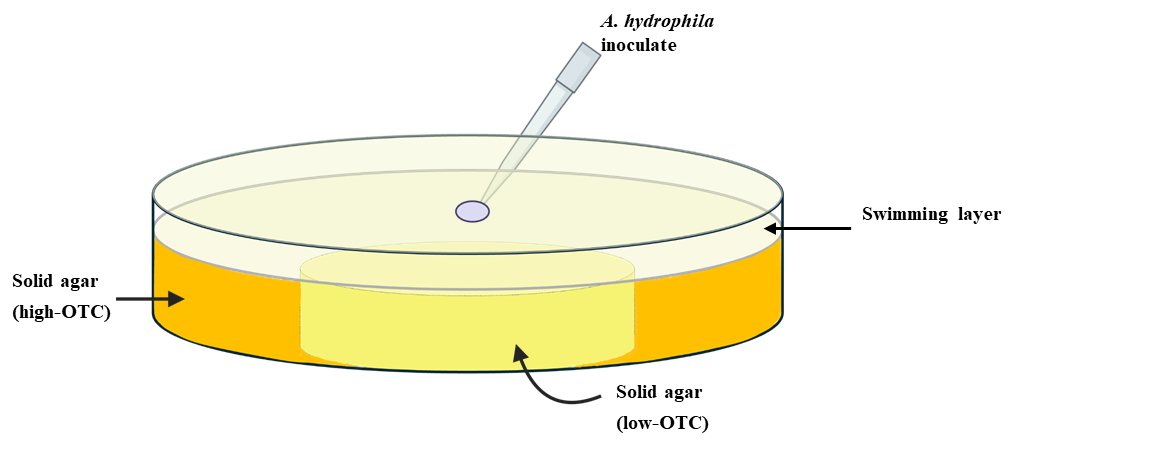


**Supplementary Figure 1**. Schematic of antibiotic resistance training procedure. The base layer of the training plates (two-step gradient system) contains five OTC incremental concentrations. Xa and Xb mark the sampling sites used for HPLC-DAD analysis of the swimming layer.


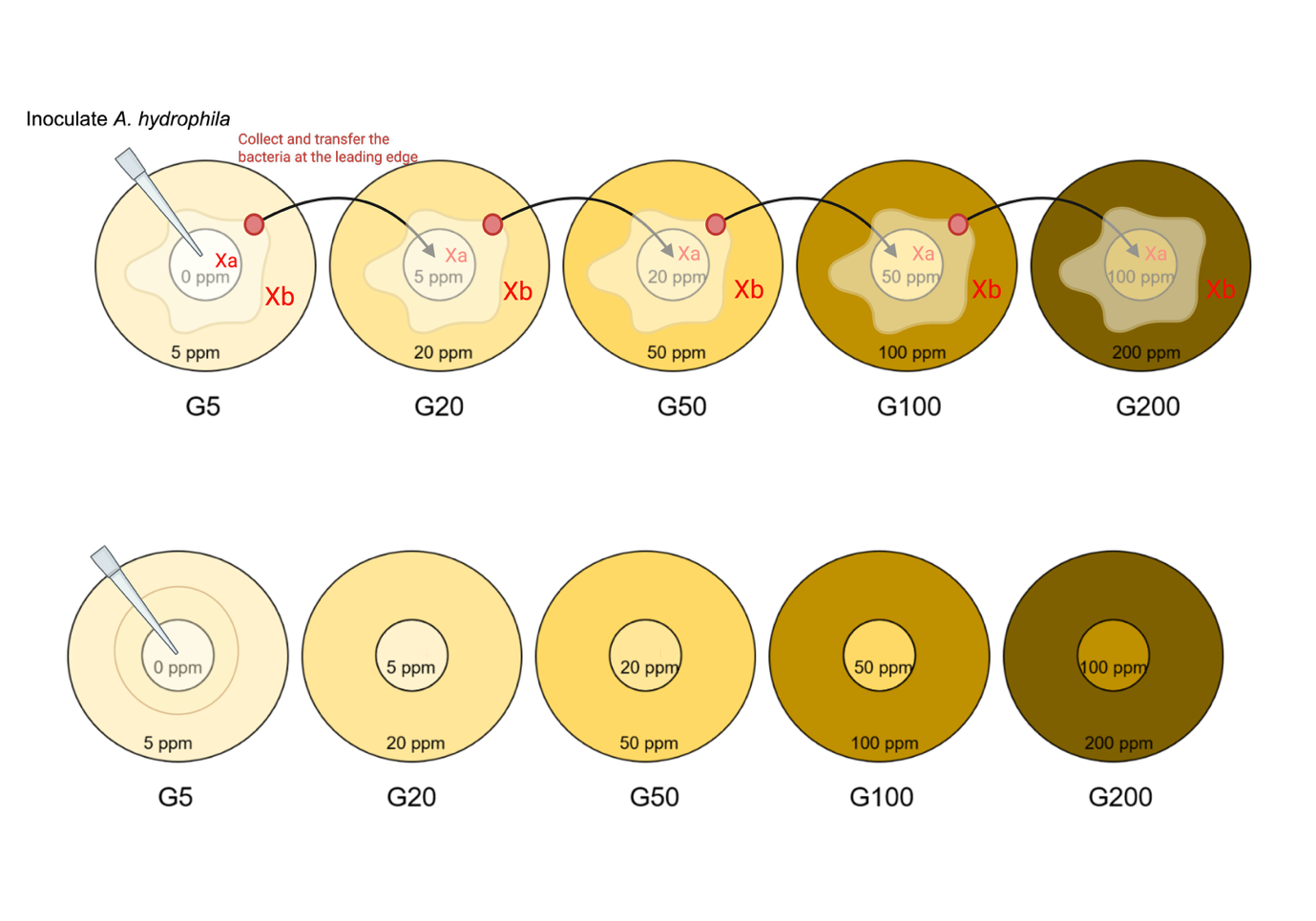

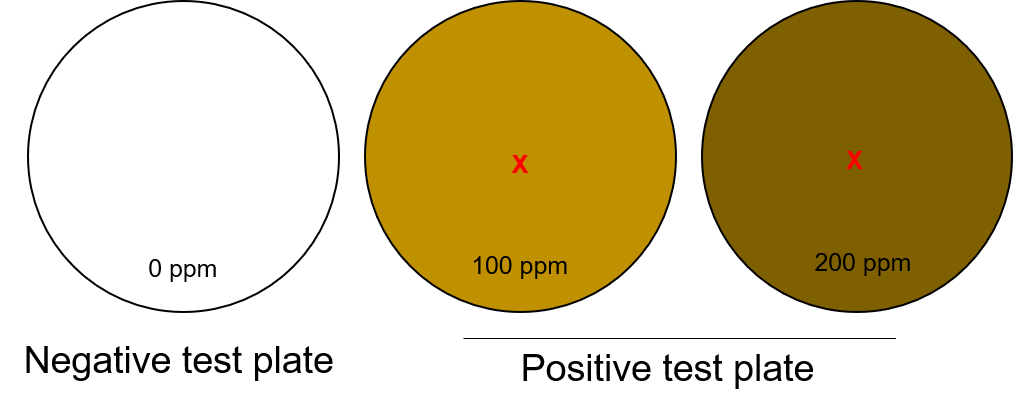


**Supplementary Figure 2.** Schematic of the test plates. The position of the X was the sampling site for HPLC-DAD analysis.
